# Duration of COVID-19 mRNA Vaccine Effectiveness against Severe Disease

**DOI:** 10.3390/vaccines10071036

**Published:** 2022-06-28

**Authors:** Devendra Bansal, Jazeel Abdulmajeed, Maha H. M. A. Al-Shamali, Soha S. A. Albayat, Sayed M. Himatt, Farhan S. Cyprian, Tawanda Chivese, Jesha M. A. Mundodan, Hayat S. Khogali, Rekayahouda Baaboura, Anvar H. Kaleeckal, Mujeeb C. Kandy, Ali Nizar Latif, Mohamed Ghaith Al-Kuwari, Hamad Eid Al-Romaihi, Abdullatif Al Khal, Roberto Bertollini, Mohamed Hamad Al-Thani, Elmobashar Farag, Suhail A. R. Doi

**Affiliations:** 1 Ministry of Public Health, Ras Qertas Street, Doha 26555, Qatar; dbansal@moph.gov.qa (D.B.); malshamali@moph.gov.qa (M.H.M.A.A.-S.); salbayat@moph.gov.qa (S.S.A.A.); shimatt@moph.gov.qa (S.M.H.); jmundodan@moph.gov.qa (J.M.A.M.); hkhogali@moph.gov.qa (H.S.K.); rekayahouda.baaboura@moph.gov.qa (R.B.); halromaihi@moph.gov.qa (H.E.A.-R.); rbertollini@moph.gov.qa (R.B.); malthani@moph.gov.qa (M.H.A.-T.); eabdfarag@moph.gov.qa (E.F.); 2 Primary Health Care Corporation, Al Mina Street, Doha 26555, Qatar; jabdulmajeed@phcc.gov.qa (J.A.); mckandy@phcc.gov.qa (M.C.K.); malkuwari@phcc.gov.qa (M.G.A.-K.); 3 Department of Population Medicine, College of Medicine, QU Health, Qatar University, University Street, Doha 2713, Qatar; tchivese@qu.edu.qa; 4 Immunology Division, Department of Basic Medical Sciences, College of Medicine, QU Health, Qatar University, Arab League Street, Doha 2713, Qatar; fcyprian@qu.edu.qa; 5 Hamad Medical Corporation, Doha 3050, Qatar; akaleeckal@hamad.qa (A.H.K.); alatif2@hamad.qa (A.N.L.); aalkhal@hamad.qa (A.A.K.)

**Keywords:** COVID-19, mRNA vaccine, waning, vaccine effectiveness, conditional effectiveness, case-control

## Abstract

Waning immunity following administration of mRNA-based COVID-19 vaccines remains a concern for many health systems. We undertook a study to determine if recent reports of waning for severe disease could have been attributed to design-related bias by conducting a study only among those detected with a first SARS-CoV-2 infection. We used a matched case-control study design with the study base being all individuals with first infection with SARS-CoV-2 reported in the State of Qatar between 1 January 2021 and 20 February 2022. Cases were those detected with first SARS-CoV-2 infection requiring intensive care (hard outcome), while controls were those detected with first SARS-CoV-2 infection who recovered without the need for intensive care. Cases and controls were matched in a 1:30 ratio for the calendar month of infection and the comorbidity category. Duration and magnitude of conditional vaccine effectiveness against requiring intensive care and the number needed to vaccinate (NNV) to prevent one more case of COVID-19 requiring intensive care was estimated for the mRNA (BNT162b2/mRNA-1273) vaccines. Conditional vaccine effectiveness against requiring intensive care was 59% (95% confidence interval (CI), 50 to 76) between the first and second dose, and strengthened to 89% (95% CI, 85 to 92) between the second dose and 4 months post the second dose in persons who received a primary course of the vaccine. There was no waning of vaccine effectiveness in the period from 4 to 6, 6 to 9, and 9 to 12 months after the second dose. This study demonstrates that, contrary to mainstream reports using hierarchical measures of effectiveness, conditional vaccine effectiveness against requiring intensive care remains robust till at least 12 months after the second dose of mRNA-based vaccines.

## 1. Introduction

The Pfizer-BioNTech (BNT162b2) and Moderna (mRNA-1273) mRNA-based vaccines are given in two doses scheduled three to four weeks apart. Evidence is still accruing regarding the duration and magnitude of protection afforded by these two mRNA vaccines. While antibody kinetics have suggested that effectiveness against any infection may decline over time [1], this may ignore the presence of non-serologic components of the immune response. Subsequent studies concur with the concern over waning of both antibody titers and vaccine effectiveness against any infection over time, especially among older populations [2,3,4]. This is more of a problem with protection against any infection (rather than severe disease), and Pfizer-BioNTech reported a gradual decline in efficacy from 96% between 7 days and 2 months, to 84% between 4 and 6 months for infection; however, they also reported that efficacy was 97% for severe disease during this period [5].

Findings regarding severe disease from several studies concur with the Pfizer-BioNTech report, and vaccine effectiveness against hospitalization and/or severe disease is reported to range from 84–96%, up to 6 months following vaccination [4,6,7,8]. However, these studies did not exclude the waning of immunity against severe disease, and more recent studies [9,10] on vaccine effectiveness suggested varying recommendations on the timing of a booster (third) dose. In this study, we address the potential of bias due to previous effectiveness study designs by undertaking an evaluation of those detected with COVID-19 in the State of Qatar, with and without the requirement for intensive care, between 1 January 2021 and 20 February 2022 to assess by how much the risk of requiring intensive care is decreased if someone does get detected with infection (conditional vaccine effectiveness) [11].

## 2. Methods

### 2.1. Design and Reporting

A matched case-control design was used, with the study base being all individuals with first infection (irrespective of vaccination status) with SARS-CoV-2 reported in the State of Qatar between 1 January 2021 and 20 February 2022. Case participants were those detected with infection requiring intensive care, and control participants were those detected with infection who recovered without the need for intensive care (see section on *COVID-19 Testing Data* below for details on how people with first infection were chosen for testing and detection).

Cases and controls were matched in a 1:30 ratio for the calendar month of infection and comorbidity category, with an exact match used. Non-pharmaceutical interventions (NPI) were mandated in the State of Qatar during the study period and included masks and social distancing with varying levels of restrictions over time. However, given matching by calendar month of infection, there was no expected impact of such temporal trends on this study. This study was reported according to the Strengthening the Reporting of Observational Studies in Epidemiology (STROBE) checklist (Table S1), and recommended additional elements for reporting COVID-19 vaccine effectiveness studies [12].

### 2.2. Data Sources

Demographic information and clinical characteristics data were obtained from the Ministry of Public Health (MoPH), Doha, Qatar, Surveillance and Vaccine Electronic System (SaVES). The MoPH database contains demographic and comorbidity information on all persons residing in Qatar who tested positive for SARS-CoV-2. The comorbidities include a range of chronic conditions (diabetes, hypertension, other cardiovascular diseases, asthma/COPD, cerebrovascular disease, rheumatological diseases, cancer, kidney disease, neurological disease, hematological disorders, immunity-related disorders, liver disease and obesity), and this was classified as none, 1 to 4, and > 4 conditions for this study. Age in years was extracted and modeled as a continuous variable (see statistical methods). 

This database receives reverse transcription-polymerase chain reaction (PCR) confirmed case notification from Hamad Medical Corporation (HMC), which is the main non-profit health care provider that manages ten highly specialized hospitals. Further links were made to intensive care admissions data retrieved from the electronic medical record at HMC and vaccination data retrieved from the Primary Health Care Corporation (PHCC), which runs 28 country-wide health centers. The vaccination data included the vaccine types and dates of the first and second dose of the two-dose vaccine schedule, as well as the date of a third dose if administered (commenced in September 2021 in Qatar). These linked databases constituted the national federated databases for COVID-19 in Qatar.

### 2.3. Vaccination

All members of the population vaccinated in Qatar received one of the two mRNA vaccines as the primary two dose schedule three (BNT162b2) to four (mRNA-1273) weeks apart. Very few participants (<1%) did not come back for the second dose. Vaccination commenced on 21 December 2020 and the booster (third dose) commenced in September 2021. The same brand of mRNA vaccine was used in the booster as in the primary series in the majority of the population. As of 20 February 2022, a total of 1,493,005 persons received at least one dose of BNT162b2, and 1,485,811 completed the two doses, while 1,012,309 persons received at least one dose of mRNA-1273, and 1,002,969 completed the two doses. Very few residents (3%) also received the AstraZeneca (ChAdOx1) vaccine as well. For the booster dose of mRNA-1273, half the dose used in the primary series was administered. Booster doses were initially administered 8 months after the second dose, but later this was reduced to 6 months because of concerns regarding possible waning of protection from the primary schedule. All participants in the date range of the study, vaccinated or not, were included if they met the inclusion criteria (first positive notification and, if vaccinated at the time of first positive notification, had completed or went on to complete the primary schedule). Finally, first infection was categorized into seven intervals in relation to the second dose of the primary vaccine schedule as follows: 0—infected when unvaccinated or infected prior to the first dose of the vaccine; 1—infected in the period between the first and second dose; 2—infected after the second dose and till four months (day 119) after the second dose; 3—infected four to six months (day 179) after the second dose; 4—infected six to nine months (day 269) after the second dose; 5—infected nine to twelve months (day 391) after the second dose; 6—infected after the third (booster) dose (data available from September 2021 to February 2022). Partially vaccinated participants (one dose only) were excluded from this categorization.

### 2.4. COVID-19 Testing Data

Nasopharyngeal and/or oropharyngeal swab collection for real-time PCR testing is carried out at HMC, PHCC, and other governmental, semi-governmental, and private health institutions across the country. Collected swabs were placed in Universal Transport Medium (UTM) and the PCR tests for SARS-CoV-2 in Qatar were undertaken by the HMC and details regarding the laboratory methods have been published previously [9,13]. More recently, rapid antigen tests were also introduced for testing at health care facilities on or after 5 January 2022, but very few infected participants had only the rapid antigen test (0.09%) and thus were not analyzed separately. Testing was available to anyone with new continuous respiratory symptoms or anyone who was a contact of a person with a confirmed case. Tests performed also include random samples tested for surveillance purposes, pre- and post-travel tests and individual test requests. Data on the *first positive test* for those tested were extracted from all tests conducted during 1 January 2021 up to 20 February 2022. 

### 2.5. Statistical Analysis

Descriptive statistics were used to describe the first-time positive SARS-CoV-2 participants selected into the matched case-control study. Time interval of this positivity (first infection) in relation to the vaccination schedule was included as an independent variable, and effectiveness was assessed using a conditional logistic regression model. Vaccine effectiveness was defined as 1 minus adjusted odds ratio of requiring intensive care in each of the time periods amongst those detected with first infection. Of note, this is considered conditional vaccine effectiveness and it is much more informative since it conveys “*how much the risk of requiring intensive care is decreased if someone does get detected with infection*” [11]. The main confounders were age, calendar month of infection and comorbidity group, with the latter two being matched for. Vaccine effectiveness was adjusted in the conditional logistic regression model for age (continuous in years modeled using restricted cubic splines with four knots). Secondary analyses were not possible for the type of vaccine (BNT162b2, or mRNA-1273 vaccine) as the majority received the BNT162b2 vaccine and data was sparse when thus stratified. Ethnicity of the person (Qatari or non-Qatari) or gender were not considered confounders as an independent association with time in relation to vaccination is unlikely given the equal access to health care for all residents of Qatar; therefore, these variables were not considered further. 

Age specific absolute risk reduction (ARR) was computed using effectiveness results from the conditional logistic regression and age-specific baseline risk (of requiring intensive care) estimated from the whole unvaccinated population (that is, all SARS-CoV-2 positive participants and not only those selected into the case control study), and used to derive a second estimate of vaccine effectiveness—the number needed to vaccinate (NNV) to prevent one more case of COVID-19 requiring intensive care. This is computed as 1/ARR and provides a different perspective because the latter combines vaccine effectiveness with the background risk of requiring intensive care. The main driver of background risk is patient age, the latter being the most critical determinant of the risk of requiring intensive care [14,15]. 

No exclusions were made for the AstraZeneca vaccine as its frequency was too small to influence assessment of the mRNA vaccines, but a sensitivity analysis was carried out after exclusion of those individuals. Goodness of link was assessed via the linktest in Stata, and goodness of fit of the model was assessed using McFadden’s R^2^, where 0.2 to 0.4 represents an excellent fit [16]. All analyses were conducted using Stata Version 15, College Station, TX, USA. 

### 2.6. Ethics

Approval and consent to participate were obtained (ethics approval ERC-826-3-2020), and waiver of informed consent was given by the Health Research Governance Department at the Ministry of Public Health. All data were de-identified before sharing for analysis.

## 3. Results

### 3.1. Descriptive Characteristics

**Figure 1:**
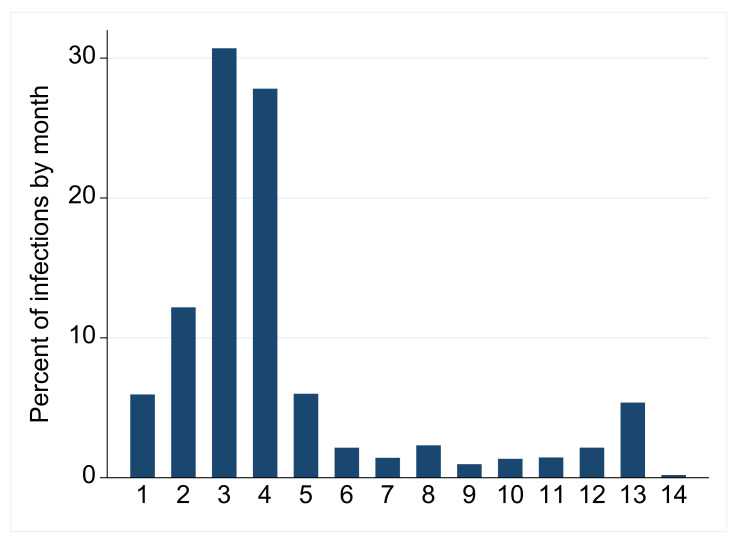
Percentage of cases by month among the participants of the case-control study. The two major waves are depicted over the 14-month study period (1–12 are January–December 2021, and 13–14 are January–February 2022).

The entire cohort of first infections reported between 1 January 2021 and 20 February 2022 in the State of Qatar were the study base of this matched case-control study. Matching (calendar month and comorbidity group) was not successful for 89 cases (4.1%), and the rest received 3–30 matched controls with 2089 (95.3%) having 30 matched controls, and a total of 64,973 participants were generated (henceforth study participants). Of the study participants, 76.3% had first infection before vaccination, 22.5% during or after the primary dose schedule, and 1.2% after the third (booster) dose; 2102 progressed to require intensive care. The distribution of the individuals in the matched case-control study (stratified by case and control status) in relation to age, sex, time interval (in relation to vaccination), comorbidity category, and vaccine type is reported in Table 1. Among these study participants, median interval between the first and second dose was 21 days (IQR 21–28) for the BNT162b2 vaccine, and 28 days (IQR 28–35) for the mRNA-1273 vaccine. The median interval from the second to the third (booster) dose among the study participants was 239 days (IQR 210–264). The median interval between the third dose and detection of the first infection among study participants was 46 days (IQR 21–82.5). The predominant circulating variants in the wave in March–April 2021 were the B.1.1.7 (or alpha) and B.1.351 (or beta) variants [4], while in the December 2021–January 2022 wave, B.1.1.529 (omicron) was the variant (Figure 1). 

### 3.2. Vaccine Effectiveness

**Figure 2:**
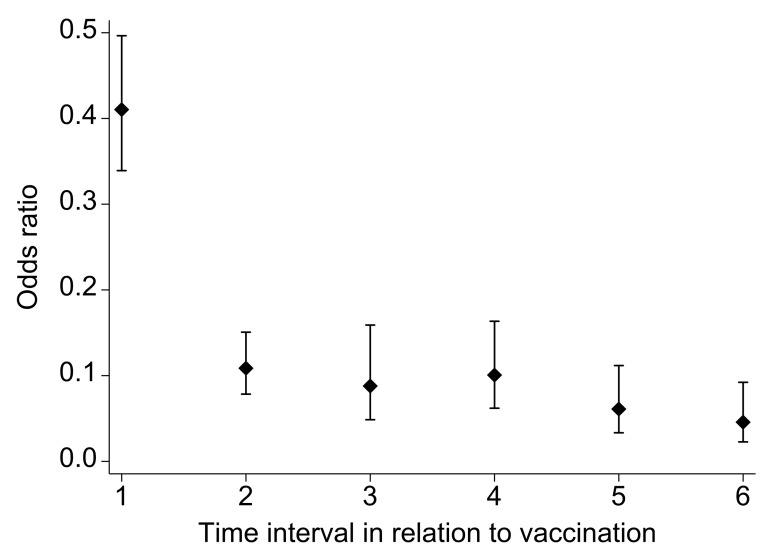
Adjusted odds ratios (1-VE) of requiring intensive care by interval in relation to vaccination from the matched case-control study Note that these results account for temporal trends in variants, NPI and changes in incidence (waves) or susceptible population over time by design (matching by calendar period). Time intervals: 0 = reference group infected prior to vaccination; 1 = those infected between first and second dose; 2 = those infected between the second dose to four months later; 3 = those infected between the fourth to sixth month after the second dose; 4 = those infected between the sixth to ninth month after the second dose; 5 = those infected between the ninth to twelfth month after the second dose; 6 = those infected after the third dose.

There were 64,259 (98.9%) participants that had non-missing data needed for the adjusted vaccine effectiveness model, from which 58 groups (1705 observations) were dropped because of all positive or all negative outcomes. Vaccine effectiveness against requiring intensive care in persons who received a primary course of the BNT162b2 or mRNA-1273 vaccine according to time interval in relation to the primary immunization schedule is reported in Figure 2. Vaccine effectiveness was 59% (95% confidence interval (CI), 50 to 76) between the first and second dose, and strengthened to 89% (95% CI, 85 to 92) between the second dose and 4 months post the second dose. Vaccine effectiveness remained at this level (91%; 95% CI 84 to 95)) between 4 and 6 months after the second dose, at 6–9 months after the second dose (90%; 95% CI, 84 to 94), and at 9–12 months after the second dose (94%; 95% CI, 89 to 97). After the third dose (booster vaccine), effectiveness had strengthened to 95% (95% CI, 91 to 98). Goodness of link and fit of the regression model were both assessed to be satisfactory. There was no appreciable difference in vaccine effectiveness results when the case-control study was built and analyzed after the exclusion of individuals who had taken the AstraZeneca vaccine. 

As expected, risk of COVID-19 requiring intensive care in the study participants was dependent on the patients age and the NNV increased from 178 at age sixty (estimated unvaccinated risk of requiring intensive care 0.6%) to 1183 (estimated unvaccinated risk of requiring intensive care 0.09%) at age thirty in the interval between nine and twelve months of the second dose. 

## 4. Discussion

This study demonstrates that conditional vaccine effectiveness for severe COVID-19, compared to estimates of hierarchical vaccine effectiveness, does not drop between 1 month and 12 months after the second vaccine dose of the primary vaccine schedule. This contrasts with a recent meta-analysis [17] that included 12 studies evaluating hierarchical vaccine efficacy or effectiveness over time for severe COVID-19 that reported that an average decrease by 10.0 percentage points (95% CI 6.1–15.4) among people of all ages, and by 9.5 percentage points (5.7–14.6) among older people between 1 month and 6 months after the final vaccine dose. Two recent studies that have tried to shed light on the effectiveness against severe disease have demonstrated the same trend, and the first one used a matched cohort design suggesting possible waning of hierarchical effectiveness by a reduced estimated hazard ratio of severe disease after a booster dose compared to no booster dose administered 6 to 8 months after the primary series [9]. The second study used a prospective cohort design and concluded that hierarchical vaccine effectiveness remains high 5 to 8 months after the primary series, but at the same time suggested that there may be waning protection against severe disease because effectiveness was greater 0 to 3 months after a booster compared to 0 to 3 months after the second dose [10]. In contrast, in this study we evaluated conditional effectiveness at up to 12 months after the primary series against the requirement for intensive care, and this remained consistent at 89–94%.

The decrease in vaccine efficacy or effectiveness over time for severe disease reported in the literature are unlikely to be variant-related [17], and we suggest that this instead may be design-related. The observational designs used previously include mainly test-negative design case-control studies and retrospective or prospective cohort studies, and these are prone to unmeasured biases [17,18] such as temporal trends for people who are vaccinated earlier being at sustained increased risk of infection compared with those who were vaccinated later, change in behavior after vaccination, temporal changes in testing frequency over time and differences in infection-derived immunity in the unvaccinated that may all lead to greater reductions in vaccine efficacy or effectiveness. This study avoided many of these biases by examining a complete consecutive cohort of COVID-19 cases in the State of Qatar in a defined period, and then examining vaccine effectiveness in terms of progression to severity. Temporal differences in dominant SARS-CoV-2 variants were accounted for by matching for calendar month. In addition, comorbidities are difficult to model because of collinearity with age, and were therefore matched by group in this study. The key remaining confounder in this design was age, and this was dealt with robustly within the analysis using continuous age and restricted cubic splines for expected non-linearity. The handling of age has been a major issue in previous studies as matching on 10-year age groups [9] or using a binary age cutoff at 55 years [10], for example, may result in significant residual confounding because age is a very critical factor in progression to severe disease requiring intensive care [9,10]. Indeed, this has led to the reported effectiveness to vary by age group in some studies with broad age groupings [10,19]. Finally, none of the previous studies looked at conditional vaccine effectiveness, which considers that outcomes of hierarchically increasing severity may be subsets of each other [11]. 

The conditional vaccine effectiveness results are backed up by immunological data. First, mRNA vaccines result in the early production of serum IgA, IgM and IgG antibodies [20,21], and this accounts for the early effectiveness for infection at least 14 days following the first dose [22]. While vaccine effectiveness against SARS-CoV-2 infection tends to decline with declining antibody titres [4,23,24,25,26,27,28], these studies failed to report the levels of neutralizing antibodies and T cell responses directed towards SARS-CoV-2 that are paramount in conferring longer term protection [1], especially against progression to severity. Other studies have demonstrated that vaccination induces long-lasting memory B and T cell responses [29,30,31]. This is consistent with the observation that natural infection with SARS-CoV-2 leads to a robust adaptive memory response that remains fairly constant 6–12 months post-infection [32]. In addition, most studies attempt to examine memory immune response to SARS-CoV-2 in peripheral blood from donors that typically lack the memory T and B cell repertoire, while abundant SARS-CoV-2 reactive memory T and B cells reside in pulmonary lymph nodes with active germinal centers harboring SARS-CoV-2 specific follicular T helper cells that persist at least 6 months after resolution of infection [33]. The presence of T follicular helper cells in germinal centers indicates active affinity maturation with diverse antibody production conferring enhanced protection [33,34]. Indeed, publications from our group [35,36] and several others have shown a direct correlation of decreased lymphocyte count with COVID-19 severity and mortality, while higher lymphocyte counts confer protection [37,38,39]. Interestingly, increased pro-inflammatory myeloid cells in the lung tissue and peripheral blood correlate with mortality and age [40]. It is likely therefore that the sustained protection against requiring intensive care results from a sustained adaptive memory response.

## 5. Conclusions

To conclude, we demonstrate that effectiveness against requiring intensive care in those detected with a first infection with SARS-CoV-2 is sustained at 89–94% until at least 12 months after the second dose with no evidence of waning, and this design that we used mitigates concerns related to the biases discussed above. We also report here the NNV for those aged sixty being 178, and those aged thirty being 1183, and this gives more information than just relative odds reductions that can then affect the interpretation of vaccine effectiveness for policy makers [41]. The NNV is strongly age-dependent, as we demonstrate here, and therefore just looking at the relative summary measure for effectiveness fails to put the effectiveness results in context. In other words, the most vulnerable groups have lower NNVs, given that their baseline risk of severe disease is larger. This study supports the conclusion that a booster shot at 12 months can be a reasonable policy decision since, for detected infections, subsequently requiring intensive care is the main burden on health systems and the main source of mortality. Future studies should report age specific NNVs in addition to vaccine effectiveness or efficacy over time, and extend follow-up beyond 12 months, as these are the outcomes that will help consolidate COVID-19 policy decisions. 

## Figures and Tables

**Table 1:** Baseline characteristics of the participants included in the matched case control study.

Factor	Level	Normal Care	Intensive Care
N		62,871	2102
Gender			
	Female	22,192 (35.3%)	568 (27.0%)
	Male	40,679 (64.7%)	1534 (73.0%)
Age (years)	Mean (SD)	36.4 (15.0)	50.4 (14.8)
Calendar month (matched)	Median (IQR)	4.0 (3.0, 4.0)	4.0 (3.0, 4.0)
Time of infection			
	*Before vaccination or not vaccinated*	47,237 (75.9%)	1763 (86.3%)
	*Between doses I and II*	5493 (8.8%)	126 (6.2%)
	*After dose II till < 4 months*	4309 (6.9%)	41 (2.0%)
	*Between 4 and < 6 months after dose II*	1133 (1.8%)	15 (0.7%)
	*Between 6 and < 9 months after dose II*	2110 (3.4%)	51 (2.5%)
	*Between 9 and 12 months after dose II*	1193 (1.9%)	32 (1.6%)
	*After the third dose*	740 (1.2%)	16 (0.8%)
Vaccine type			
	BNT162b2 (30 µg)	32,785 (64.6%)	814 (61.6%)
	mRNA-1273	17,277 (34.0%)	490 (37.1%)
	BNT162b2 (10 µg)	104 (0.2%)	0
	ChAdOx1 (AstraZeneca)	602 (1.2%)	18 (1.4%)
Comorbidity (matched)			
	None	34,110 (54.3%)	1137 (54.1%)
	1–4	27,674 (44.0%)	924 (44.0%)
	> 4	1087 (1.7%)	41 (2.0%)

## Data Availability

The dataset of this study is held at the Ministry of Public Health, Qatar, and was accessed by the researchers through a restricted-access agreement that prevents its sharing with a third party or publicly.

## Supplementary Materials

The following are available online at https://www.mdpi.com/article/10.3390/vaccines10071036/s1, Table S1: Strengthening the Reporting of Observational Studies in Epidemiology (STROBE) checklist and recommended additional elements for reporting COVID-19 vaccine effectiveness studies.
