# Supplementary material for: Duration of COVID-19 mRNA Vaccine Effectiveness against Severe Disease
Source: Vaccines (Basel). 2022 Jun 28;10(7):1036. doi: 10.3390/vaccines10071036 (PMC9321581; doi:10.3390/vaccines10071036)
Supplement: Supplementary file 1 [file vaccines-10-01036-s001.zip › vaccines-1751546-supplementary.pdf]

STrengthening the Reporting of OBservational studies in Epidemiology (STROBE) checklist with recommended additional elements for reporting COVID-19 vaccine effectiveness studies.

SECTION/TOPIC	STROBE ITEM NO	STROBE	COVID-19 VE STUDIES	REF PAGE*
**TITLE AND ABSTRACT**				
TITLE/ABSTRACT	1	Indicate the study's design with a commonly used term in the title or the abstract	Specify study design (e.g., case-control, TND or cohort)	1
		Provide in the abstract an informative and balanced summary of what was done and what was found	Report vaccine type(s), outcome, target vaccine groups evaluated, study location, VE and 95% confidence intervals	1
**INTRODUCTION**				
BACKGROUND/ RATIONALE	2	Explain the scientific background and rationale for the investigation being reported	Mention efficacy results from pivotal clinical trial that led to EUL/EUA or licensure of vaccine being studied	2
			Describe specific vaccine products in use, timeline of introduction, targeted populations and coverage, NPI measures in place in study area	4
			Describe COVID-19 epidemiology preceding and during period of study, including baseline seroprevalence in the target population if known, disease activity, and predominant variants during the study	4, Figure 2
OBJECTIVES	3	State specific objectives, including any prespecified hypotheses	Was study done to provide local/subpopulation VE estimates or answer global evidence gap in VE data?	2
**METHODS**				
STUDY DESIGN	4	Present key elements of study design early in paper	TND, traditional case-control, cohort, other	2
SETTING	5	Describe the setting, locations, and relevant dates, including periods of recruitment, exposure, follow-up, and data collection	Describe the enrollment setting (e.g. SARI surveillance, hospitalized patients), location or region<br>COVID-19 incidence at time of study, vaccines in use, introduction dates, and timing of rollout in target groups, NPI measures in place, and common circulating SARS-CoV-2 variants	2
			Report time period when data were collected	2
PARTICIPANTS	6	Cohort study—Give the eligibility criteria, and the sources and methods of selection of participants. Describe methods of followup.<br>Case-control study—Give the eligibility criteria, and the sources and methods of case ascertainment and control selection. Give the rationale for the choice of cases and controls	Report specific clinical case definition used for enrollment	2
			Report definition of severity used	3
			Describe eligible study population in terms of age and vaccine target groups (e.g., HWs, chronic medical conditions) and exclusion criteria	4
VARIABLES	7	Clearly define all outcomes, exposures, predictors, potential confounders, and effect modifiers. Give diagnostic criteria, if applicable	COVID-19 vaccine variables<br>Report definition for vaccination status, including exclusions based on vaccine timing (e.g., receipt of vaccine < 14 days of illness onset) and fully vs. partially vaccinated, dose interval COVID-19 outcomes	3
			Report sensitivity and specificity of diagnostic test used; if rapid antigen test, give test name and antigen target;	N/A as RT-PCR has known Se and Sp
			Indicate if COVID-19 result known prior to or after enrollment. Explain how possible vaccine reactions were handled in TND studies (e.g., exclude recent vaccinees tested for possible febrile reaction to vaccine) Covariates	2
			Report covariates assessed for confounding, and if and how adjusted for	4

SECTION/TOPIC	STROBE ITEM NO	STROBE	COVID-19 VE STUDIES	REF PAGE*
			Report the specific cut points used for continuous variables that are categorized (e.g. age groups).	2,3
			Provide the list of conditions included as ‘‘high risk”	2
			Provide the unit of time if adjusting for calendar time.	2
			Describe how prior COVID-19 infection was defined	N/A
DATA SOURCES/ MEASUREMENT	8	For each variable of interest, give sources of data and details of methods of assessment (measurement).	*COVID-19 vaccine* Report source of vaccination data (e.g., vaccine card, medical record, registry, provider report, patient report, or some combination of the above).	2
		Describe comparability of assessment methods if there is more than one group	List the type and brand of vaccine (lot number if available).	3
			Report recommended schedule for vaccination (number of doses and time interval between doses)	3
			COVID-19 outcomes Report procedures for collection of respiratory samples and RT-PCR testing, include type of respiratory samples collected (e.g. nasal, nasopharyngeal), type of swab used (e.g. flocked), transport media (e.g. universal transport media or report if dry swabs were used) and maximum interval from onset to swab collection;	3
			Report up to how many days before enrollment a positive COVID-19 test was acceptable; Were subjects with compatible clinical illness without lab confirmation enrolled?	N/A
BIAS	9	Describe any efforts to address potential sources of bias	Report if prior COVID-19 infection and exposure risk to COVID-19 (e.g., mask-wearing) were assessed and how handled	N/A
STUDY SIZE	10	Explain how the study size was arrived at	Adjust sample size calculation to expected COVID-19 incidence and estimated VE from clinical trial	N/A
QUANTITATIVE VARIABLES	11	Explain how quantitative variables were handled in the analyses. If applicable, describe which groups were chosen and why	Report the specific cut points used for continuous variables that are categorized (e.g. age groups). Provide the unit of time if adjusting for calendar time	2
STATISTICAL METHODS	12	Describe all statistical methods, including those used to control for confounding	Describe the specific regression method used (e.g. logistic regression) and confidence limits methodology	3
			Report the time periods for which data were analyzed and if COVID-19 was circulating throughout	2
			Specify any matching variable (e.g. time) and whether regression model accounts for matching	2
			Specify how covariates assessed for inclusion in the model and final covariates included	4
			Describe how partially vaccinated persons were handled in the analysis (e.g., one dose)	3
			Describe how data were pooled if gathered from multiple sites and measure of heterogeneity calculated	N/A
STATISTICAL METHODS	12	Describe any methods used to examine subgroups and interactions	Describe any analyses of subgroups (e.g. age groups, chronic conditions, HWs)	4, Table 1
			Describe interactions assessed (e.g. prior COVID-19 infection)	N/A
		Explain how missing data were addressed	Describe whether a complete case analysis was used or if missing data were imputed. Name	4

SECTION/TOPIC	STROBE ITEM NO	STROBE	COVID-19 VE STUDIES	REF PAGE*
			the package used for imputation (e.g. ICE in Stata).	
		Cohort study—If applicable, explain how loss to follow-up was addressed<br>Case-control study—If applicable, explain how matching of cases and controls was addressed	In case-control studies, if more than one control group enrolled, explain rationale.	N/A
		Describe any sensitivity analyses	For example, excluding verbal reports of vaccination; limited to positive test within 72 h of enrollment; limited to PCR + only (if rapid antigen tests included)	4
OTHER			Indicate if and where study protocol and/or study data are publicly available	7
**RESULTS**				
PARTICIPANTS	13	a) Report numbers of individuals at each stage of study— e.g., numbers potentially eligible, examined for eligibility, confirmed eligible, included in the study, completed follow-up, and analyzed		4
		b) Give reasons for non-participation at each stage		N/A
		c) Consider use of a flow diagram		
DESCRIPTIVE DATA	14	a) Give characteristics of study participants (e.g., demographic, clinical, social) and information on exposures and potential confounders	Describe percentage of each COVID-19 vaccine used in the study population	Table 1
			Report number of participants who received only one dose of two dose schedule, and if different vaccines given for each dose	4
			Describe seroprevalence of study population, if available	N/A
		b) Indicate number of participants with missing data foreach variable of interest		N/A
		c) Cohort study—summarize follow-up time (e.g., average and total amount)		N/A
OUTCOME DATA	15	Cohort study—Report numbers of outcome events or summary measures over time<br>Case-control study—Report numbers in each exposure category, or summary measures of exposure	Describe number/percent of tests which were PCR, rapid antigen test, other.	Table 1
			Report COVID-19 genomic information among vaccine failures, if available. Particularly variants of concern.	N/A
MAIN RESULTS	16	a) Give unadjusted estimates and, if applicable, confounder-adjusted estimates and their precision (e.g., 95% confidence intervals). Make clear which confounders were adjusted for and why they were included	Report adjusted VE and 95% CI by vaccine type	5
			Report adjusted VE and 95% CI for target groups separately, if sufficient power	5
			Report heterogeneity statistics for pooled data	N/A
		b) Report category boundaries when continuous variables were categorized		Table 1
		c) If relevant, consider translating estimates of relative risk into absolute risk for a meaningful time period		NNV reported
OTHER ANALYSES	17	Report other analyses done—e.g. analyses of subgroups and interactions, and sensitivity analyses	Report age-stratified VE and 95% CI estimates separately	5
			Report separate VE and 95% CI among those with one dose, two doses and at least one dose COVID-19 vaccines	5
			Report separate VE and 95% CI by SARS-CoV-2 variant if sufficient power	N/A

SECTION/TOPIC	STROBE ITEM NO	STROBE	COVID-19 VE STUDIES	REF PAGE*
**DISCUSSION**				
KEY RESULTS	18	Summarize key results with reference to study objectives		6
LIMITATIONS	19	Discuss limitations of the study, taking into account sources of potential bias or imprecision. Discuss both direction and magnitude of any potential bias	Specifically discuss potential biases affecting COVID-19 VE studies, including health-seeking bias, misclassification bias, diagnostic bias	5
INTERPRETATION	20	Give a cautious overall interpretation of results considering objectives, limitations, multiplicity of analyses, results from similar studies, and other relevant evidence	Explain potential differences in study VE from efficacy in relevant clinical trials (e.g., different target group, different outcome, immunization system factors)	5
GENERALIZABILITY	21	Discuss the generalizability (external validity) of the study results	Was baseline seroprevalence different from other settings? Predominant viral variant found in other settings?	5
**OTHER INFORMATION**				
FUNDING	22	Give the source of funding and the role of the funders for the present study and, if applicable, for the original study on which the present article is based		7

* REF PAGE- Reference page in the main manuscript file.
